# Characteristics, clinical management and outcomes of patients with acute myocardial infarction enrolled or not enrolled in a quality registry

**DOI:** 10.1093/ehjqcco/qcag023

**Published:** 2026-02-10

**Authors:** Masih Khedri, Karolina Szummer, Stefan H Jacobson, Paul Hjemdahl, Jonas Spaak, Juan-Jesus Carrero

**Affiliations:** Department of Clinical Sciences, Danderyd University Hospital, Karolinska Institutet, 182 88 Stockholm, Sweden; Medical Unit Aging, Karolinska University Hospital, 141 86 Stockholm, Sweden; Department of Medicine, Huddinge, Karolinska Institutet, 141 83 Stockholm, Sweden; Medical Unit Cardiology, Karolinska University Hospital, 141 86 Stockholm, Sweden; Department of Clinical Sciences, Danderyd University Hospital, Karolinska Institutet, 182 88 Stockholm, Sweden; Division of Nephrology, Danderyd University Hospital, 182 88 Stockholm, Sweden; Department of Medicine Solna, Clinical Epidemiology Unit, Karolinska Institutet, 171 76 Stockholm, Sweden; Medical Unit Clinical Pharmacology, Karolinska University Hospital, 141 86 Stockholm, Sweden; Department of Clinical Sciences, Danderyd University Hospital, Karolinska Institutet, 182 88 Stockholm, Sweden; Division of Cardiology, Danderyd University Hospital, 182 88 Stockholm, Sweden; Department of Clinical Sciences, Danderyd University Hospital, Karolinska Institutet, 182 88 Stockholm, Sweden; Department of Medical Epidemiology and Biostatistics, Karolinska Institutet, 171 77 Stockholm, Sweden

**Keywords:** Clinical outcomes, Guideline-recommended medical therapies, Secondary prevention, Acute coronary syndrome, Quality register

## Abstract

**Aims:**

Structured care through enrollment and data collection in quality registries may lead to better care and improved outcomes. We investigated differences in admission characteristics, clinical management and outcomes between patients with acute myocardial infarction enrolled vs. non-enrolled in the SWEDEHEART quality registry.

**Methods and results:**

We linked health records from all hospitalisations (*n* = 47 342) due to a first or recurrent myocardial infarction between 2006 and 2021 in the region of Stockholm, Sweden, to SWEDEHEART. We compared non-enrolled vs. enrolled patients in terms of characteristics, invasive procedures, use of and adherence to guideline-recommended medications, in-hospital mortality, and clinical outcomes after discharge. Non-enrolled participants (*n* = 6 113, 13%) were older, had more chronic kidney disease and other comorbidities. They underwent fewer coronary angiographies and fewer coronary interventions. Non-enrolled participants were less likely to initiate aspirin (HR 0.88, 95% CI 0.84–0.91), beta-blockers (HR 0.87, CI 0.84–0.90), renin-angiotensin system inhibitors (HR 0.73, CI 0.69–0.76), and statins (HR 0.59, CI 0.56– 0.61). They were also less likely to adhere to treatments, in part explained by their comorbid profile. Even after extensive adjustments, non-enrolled patients had higher in-hospital and long-term mortality (HR 1.15, 95% CI 1.09–1.21), and more reinfarction/stroke (HR 1.16, 95% CI 1.08–1.26) than enrolled patients.

**Conclusion:**

Patients non-enrolled in SWEDEHEART received less evidence-based care and had worse short- and long-term outcomes. This study identifies a non-negligible population in need of better care and provides support for the value of structured care models in improving patient outcomes through closer monitoring and better treatment.

## Graphical Abstract

**Graphical Abstract:**
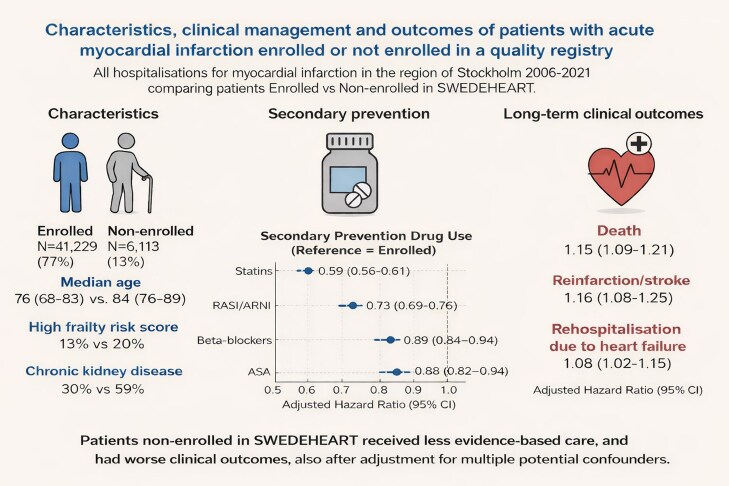

Key Learning Points What is already known

- Structured care models improve short- and long-term outcomes.
- Clinical quality registries like SWEDEHEART (Swedish Web System for Enhancement and Development of Evidence-based care in Heart disease Evaluated According to Recommended Therapies) enhance healthcare by tracking care quality and supporting research that informs guidelines.
- Registry participation may be biased due to selection factors and patient characteristics and may lead to unequal care by excluding patients who could benefit most from structured care models.

What this study adds

- About 13% of patients with myocardial infarction were not enrolled in SWEDEHEART.
- Non-enrolled patients were older and carried a higher comorbidity burden, received less evidence-based care, and had worse short- and long-term outcomes even after extensive adjustment for potential confounders.
- Our study highlights important care gaps that call for targeted interventions and support the value of structured care models in improving patient outcomes through better treatment and structured follow-up.

## Introduction

Clinical quality registries play a key role in improving healthcare by systematically tracking predefined markers of quality of care.^1-3^ In cardiology, the SWEDEHEART (Swedish Web System for Enhancement and Development of Evidence-based care in Heart disease Evaluated According to Recommended Therapies) registry is a prime example, providing comprehensive data on care and outcomes after acute myocardial infarction.^4^ Such registries not only drive quality improvement efforts but also enable research that informs clinical guidelines and practice, as an important complement to randomized clinical trials (RCTs).^2,5^ This is particularly valuable for high-risk groups such as patients with chronic kidney disease (CKD) and older adults who are frequently underrepresented in cardiovascular RCTs,^6,7^ which is a growing concern in view of the ageing cardiovascular population.^8,9^

SWEDEHEART has an extensive data collection protocol for patients with suspected acute coronary syndromes admitted to registry-affiliated wards. Over time, the registry has expanded beyond coronary care units to include some, but not all, additional hospital wards, and has gradually included older patients.^10^ On one hand, registry enrollment itself may, via structured protocols and checklists, improve the quality of care delivered. This has been seen within the SWEDEHEART registry over the years. On the other hand, registry participation is determined by whether the treating unit is affiliated with the registry, and admission to these units can depend on the patient´s conditions, thus introducing selection bias. As a result, patients enrolled in SWEDEHEART and similar registries may differ systematically from those who are not. Quantifying the numbers, quality of care and outcomes of patients non-enrolled in quality registries gives an opportunity to identify healthcare gaps amenable to intervention. Research in this area may support more inclusive and equitable quality improvement strategies that better reflect the realities of today’s ageing, multimorbid and frail patient population.

Leveraging complete electronic healthcare records from the largest region in Sweden, this study aimed to systematically examine differences in patient characteristics, clinical management, and outcomes between individuals experiencing a first or recurrent myocardial infarction who were or were not receiving structured care through enrollment in the SWEDEHEART quality registry.

## Methods

### Data sources

Data were obtained from the Stockholm CREAtinine Measurements (SCREAM) database covering all residents of Stockholm, Sweden. SCREAM captures complete health care use, including primary care and laboratory results, from January 2006 to December 2021. Using Sweden’s unique personal identification number, data were linked to the Register of Information and Knowledge About Swedish Heart Intensive Care Admissions (RIKS-HIA), a sub-registry of the SWEDEHEART registry, and to national and regional registries: socioeconomic data from Statistics Sweden (SCB), mortality from the Swedish Population Registry, prescribed drugs from the National Prescribed Drug Register (NPDR), and diagnostic and administrative codes from the Regional VAL database for all hospital admissions, specialized outpatient care and procedures. The Regional Ethical Review Board in Stockholm approved the study (EPN 2017/793-31) and waived informed consent, as all data were de-identified.

### Case identification and study exposure

All adults (≥18 years) hospitalized for a first or recurrent myocardial infarction between 2006 and 2021 were included. The hospital admission date for myocardial infarction served as the index date for covariate assessment and initiation of follow-up. Cases were identified using the ICD-10 code I21 as the primary discharge diagnosis. Linked hospitalisations with overlapping admission and discharge dates were merged into one continuous stay. Any re-hospitalisation within 30 days of discharge was attributed to the index event. Enrollment in SWEDEHEART was defined as any entry in RIKS-HIA during the index hospitalisation or one day prior to admission to capture emergency department entries. SWEDEHEART enrollment constituted the study exposure, comparing enrolled vs. non-enrolled patients.

### Patient characteristics

Covariates included sociodemographic factors, comorbidities, laboratory results, and medications. Sociodemographic variables comprised cohabitation status, highest education, and disposable income (income after tax, in quartiles). Ethnicity is not recorded in Swedish health registers due to legal restrictions; however, based on country of birth and parentage statistics, the study population is predominantly of European ancestry. To reduce bias from illness-related income changes, income from the preceding year was used. Baseline creatinine was the most recent value at admission or within 18 months prior. Estimated glomerular filtration rate (eGFR) was calculated using the CKD-EPI 2009 formula and categorized as: normal/mild (≥60 mL/min/1.73 m²), moderate CKD (30–59 mL/min/1.73 m²), or severe CKD (<30 mL/min/1.73 m²). Other laboratory values were obtained from tests during hospitalisation. Comorbidities known before admission and the type of infarction were identified through ICD-10 codes (see Supplementary material online, *Table S1*). Patients with any dispensed antidiabetic medication within 3 months were classified as having diabetes. Care in a cardiology unit or discharge from a geriatric unit were identified from administrative codes (see Supplementary material online, *Table S1*). The Hospital Frailty Risk Score was calculated and categorized based on validated algorithms.^11^ The Charlson Comorbidity Index was calculated using Quan *et al.* weights with a cut-off of ≥3.^12,13^ Patients were considered to be on treatment at admission if a prescription was dispensed within 3 months before admission (see Supplementary material online, *Table S1*).

### Clinical management

Selected quality of care indicators in routine healthcare data, focusing on invasive coronary procedures and use of key secondary prevention drugs, and informed by guideline recommendations^14-17^ were compared between groups. Invasive procedures, including coronary angiography, percutaneous coronary intervention (PCI), and coronary artery bypass grafting (CABG), were identified using procedure codes (see Supplementary material online, *Table S3*) during the index hospitalisation. Guideline-recommended discharge medications included acetylsalicylic acid (ASA), beta-blockers (BB), renin–angiotensin system inhibitors (RASi), angiotensin receptor–neprilysin inhibitors (ARNI), statins, and P2Y_12_-inhibitors (ATC codes in Supplementary material online, *Table S1*). Because prevalent users may delay pharmacy refills and prescriptions in Sweden typically cover approximately 3 months, treatment initiation or continuation was defined as any dispensed prescription within 90 days after discharge. Dual antiplatelet therapy (DAPT) was defined as prescriptions of both ASA and a P2Y_12_-inhibitor within 90 days. A 120-day cutoff distinguished DAPT ≤3 months vs. >3 months to account for package size variation. Daily dosages from NPDR text data were standardized using a semi-automated algorithm. Patients who died or were lost to follow-up within 120 days after discharge were excluded from DAPT analyses. Medication adherence was defined as a proportion of days covered (PDC) ≥ 80% among those who initiated or continued treatment within 90 days post-discharge. PDC was calculated as days covered from the first dispensed prescription until 365 days later, or until death or end of follow-up.

### Clinical outcomes

We first compared in-hospital mortality, defined as death during the index hospitalisation. Among survivors, short-term outcomes were assessed as death within the predefined 90-day post- discharge window, with covariates recalculated at discharge. In patients surviving ≥90 days, long-term outcomes included all-cause death and hospitalisations due to recurrent myocardial infarction or stroke (ICD-10 codes in primary positions) and heart failure (primary or secondary positions, Supplementary material online, *Table S1*). Follow-up continued until death, emigration, or 31 December 2021.

### Statistical analysis

Continuous variables are reported as means ± standard deviations and categorical variables as proportions. Differences between groups were tested using Student’s *t*-test and the chi-square test. Logistic regression estimated odds ratios (ORs) and 95% confidence intervals (CIs) for adherence indicators and in-hospital death. Cause-specific Cox regression estimated hazard ratios (HRs) for use of guideline-recommended drugs, censoring at death or study end. Cause-specific Cox models were also used for long-term non-fatal outcomes (reinfarction/stroke or heart failure hospitalisation), censoring at death, emigration, or end of follow-up. Cox proportional hazards models estimated HRs for short- and long-term mortality. Because individuals could experience recurrent myocardial infarction and contribute multiple observations, robust standard errors clustered at the patient level were applied in all models. Multivariable adjustments were tailored to each analysis and explained in detail in table captions and figure legends. Models of invasive procedures were adjusted for demographic factors and comorbidities. Analyses of in-hospital and short-term mortality additionally adjusted for revascularisation. Drug use and adherence were adjusted in three sequential models (see *Table 3*). Long-term outcome analyses used similar adjustments, with a fourth model including initiation or continuation of secondary preventive drugs to assess mediation through improved treatment.

Sensitivity analyses restricted to 2006–2009 and 2010–2021 admissions accounted for the increasing SWEDEHEART coverage after 2010, when enrollment substantially increased (*Table 1*). Another sensitivity analysis, limited to incident cases, tested the robustness of results without repeated observations. Supporting analyses explored subgroups at higher risk of suboptimal care, including drug use among non-enrolled patients with CKD, and long-term mortality in older adults (≥75 years) stratified by frailty risk. Interaction terms were tested for effect modification between non-enrollment and long-term mortality across frailty risk categories. Missing laboratory or socioeconomic data were coded using missing indicators. All analyses were performed using R version 4.4.2.

**Table 1:** Characteristics at admission according to quality register enrollment

Variable	All *n* = 47 342	Enrolled *n* = 41 229	Non-enrolled *n* = 6113	*P*-value
**Demographics and socioeconomic status, n, (%)**				
Male	29 962 (63%)	27 068 (66%)	2894 (47%)	<0.001
Age, years, median (IQR)	73 (63–83)	71 (62–81)	84 (76–89)	<0.001
Age category, years				<0.001
18–49	2718 (5.7%)	2636 (6.4%)	82 (1.3%)	
50–64	11 546 (24%)	11 003 (27%)	543 (8.9%)	
65–79	17 641 (37%)	16 157 (39%)	1484 (24%)	
80+	15 437 (33%)	11 433 (28%)	4004 (65%)	
Cohabitating	23 832 (50%)	21 613 (53%)	2219 (36%)	<0.001
Education level				<0.001
Compulsory school	15 148 (33%)	12 562 (31%)	2586 (45%)	
Secondary school	18 980 (42%)	16 832 (42%)	2148 (37%)	
University	11 567 (25%)	10 570 (26%)	997 (17%)	
Missing	1647 (3.5%)	1265 (3.1%)	382 (6.2%)	
Disposable Income (quartiles)				<0.001
1 (lowest)	11 807 (25%)	9972 (24%)	1835 (30%)	
2	11 805 (25%)	9859 (24%)	1946 (32%)	
3	11 804 (25%)	10 406 (25%)	1398 (23%)	
4 (highest)	11 795 (25%)	10 872 (26%)	923 (15%)	
**Laboratory variables**				
eGFR category (mL/min/1.73 m²)				<0.001
≥60	30 154 (67%)	27 795 (70%)	2359 (42%)	
30–59	11 864 (26%)	9525 (24%)	2339 (42%)	
<30	3232 (7.1%)	2296 (5.8%)	936 (17%)	
Missing	2092 (4.4%)	1613 (3.9%)	479 (7.8%)	
Any Hemoglobin <90 g/L (*n* = 43 933)	3454 (7.9%)	2866 (7.5%)	588 (11%)	<0.001
Any C-reactive protein >80 mg/L (*n* = 42 314)	9807 (23%)	8149 (22%)	1658 (32%)	<0.001
**Condition relevant to the index event**				
Infarction type				<0.001
Transmural infarction	12 028 (25%)	11 499 (28%)	529 (8.7%)	
Subendocardial infarction	24 079 (51%)	21 466 (52%)	2613 (43%)	
Unspecified	11 235 (24%)	8264 (20%)	2971 (49%)	
Calendar year				<0.001
2006–2009	13 551 (29%)	9623 (23%)	3928 (64%)	
2010–2013	11 878 (25%)	10 766 (26%)	1112 (18%)	
2014–2017	11 417 (24%)	10 821 (26%)	596 (9.7%)	
2018–2021	10 496 (22%)	10 019 (24%)	477 (7.8%)	
Care at cardiology unit	37 221 (79%)	34 620 (84%)	2601 (43%)	<0.001
Discharged to home from the geriatric unit	6279 (13%)	4506 (11%)	1773 (29%)	<0.001
**Comorbidities at admission**				
Previous acute coronary syndrome	16 722 (35%)	14 525 (35%)	2197 (36%)	0.3
Other ischemic heart disease	15 032 (32%)	12 503 (30%)	2529 (41%)	<0.001
Intracranial bleeding	827 (1.7%)	694 (1.7%)	133 (2.2%)	0.006
Gastrointestinal bleeding	1013 (2.1%)	845 (2.0%)	168 (2.7%)	<0.001
Atrial fibrillation	7606 (16%)	5929 (14%)	1677 (27%)	<0.001
AV-block of any degree	1123 (2.4%)	885 (2.1%)	238 (3.9%)	<0.001
Congestive heart failure	9997 (21%)	7554 (18%)	2443 (40%)	<0.001
Chronic obstructive pulmonary disorder	5570 (12%)	4616 (11%)	954 (16%)	<0.001
Other chronic respiratory disease	6058 (13%)	5231 (13%)	827 (14%)	0.066
Diabetes mellitus	13 264 (28%)	11 562 (28%)	1702 (28%)	0.7
Hypertension	32 313 (68%)	28 071 (68%)	4242 (69%)	0.040
Malignancy in the previous 3 years	5428 (11%)	4552 (11%)	876 (14%)	<0.001
Peripheral arterial disease	5157 (11%)	4192 (10%)	965 (16%)	<0.001
Stroke	4731 (10.0%)	3763 (9.1%)	968 (16%)	<0.001
End-stage kidney disease	501 (1.1%)	437 (1.1%)	64 (1.0%)	0.98
Dialysis (at any time)	475 (1.0%)	397 (1.0%)	78 (1.3%)	0.022
Mild cognitive impairment	2183 (4.6%)	1660 (4.0%)	523 (8.6%)	<0.001
Dementia	2327 (4.9%)	1619 (3.9%)	708 (12%)	<0.001
**Comorbidity/Frailty Risk Scores**				
Charlson Comorbidity Index ≥ 3	15 539 (33%)	12, 943 (32%)	2596 (42%)	<0.001
Hospital Frailty Risk Score				<0.001
Low Risk (<5)	23 960 (51%)	21 589 (52%)	2371 (39%)	
Intermediate Risk (5–15)	16 794 (36%)	14 285 (35%)	2509 (41%)	
High Risk (>15)	6588 (14%)	5355 (13%)	1233 (20%)	
**Medications at admission**				
Acetylsalicylic acid	16 471 (35%)	13 460 (33%)	3011 (49%)	<0.001
Other antiplatelet drug	4120 (8.7%)	3415 (8.3%)	705 (12%)	<0.001
Oral anticoagulants	3778 (8.0%)	3207 (7.8%)	571 (9.3%)	<0.001
Beta-blockers	18 882 (40%)	15 892 (39%)	2990 (49%)	<0.001
RASi/ARNI	19 964 (42%)	17 439 (42%)	2525 (41%)	0.14
Aldosterone antagonists	2554 (5.4%)	1917 (4.6%)	637 (10%)	<0.001
Statins	14 231 (30%)	12 526 (30%)	1705 (28%)	<0.001
Ezetimibe	751 (1.6%)	694 (1.7%)	57 (0.9%)	<0.001

IQR, interquartile range; AV-block, atrioventricular block; RASi, renin angiotensin system inhibitor; ARNI, angiotensin receptor-neprilysin inhibitor.

## Results

### Patient characteristics at hospital admission

**Figure 1:**
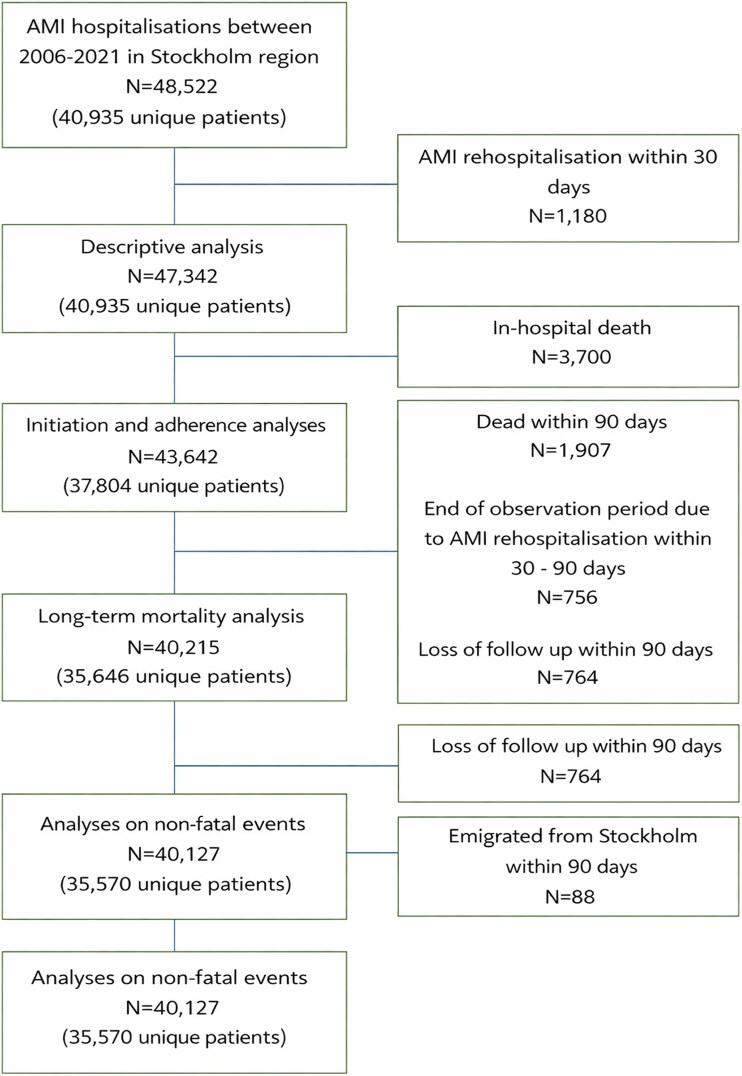
Selection of cases for descriptive analysis, assessment of drug initiation and adherence, and outcome analysis. MI: myocardial infarction.

Between 2006 and 2021, a total of 65 428 hospitalisations for first or recurrent myocardial infarction occurred in the Stockholm region, involving 40 935 unique patients. After merging linked hospitalisations and excluding re-hospitalisations within 30 days, 47 342 unique hospitalisations remained (*Figure 1*). Out of these, 41 229 participants (87.1%) were enrolled in the SWEDEHEART registry, and 6113 (12.9%) were non-enrolled. Characteristics of identified myocardial infarction registrations are presented in *Table 1*. Most non-enrolled cases (64%) occurred in the early study period (≤2009). One-third of all patients were ≥80 years. Non-enrolled patients were older, more often female, and had lower educational and income levels. CKD was present in 59% of non-enrolled vs. 30% of enrolled patients (*P* < 0.001). Non-enrolled patients also had higher prevalences of comorbidities such as atrial fibrillation, CHF, COPD, stroke, and dementia, with higher comorbidity- and frailty risk scores, but similar prevalences of diabetes and hypertension. Characteristics of unique individuals at first presentation followed a similar pattern (see Supplementary material online, *Table S2*).

### Clinical management

After adjustment, non-enrolled patients were less likely to undergo in-hospital invasive procedures such as coronary angiography or PCI (*Table 2*), with no difference for CABG. After censoring patients who died or were lost to follow-up within 90 days post-discharge, non-enrolled patients were less likely to receive ASA, BB, RASi/ARNI and statins (*Table 3*). Sensitivity analyses showed consistent results across both the early (≤2009) and later (≥2010) study periods (see Supplementary material online, *Tables S3* and *S4*), and in analyses restricted to incident cases (see Supplementary material online, *Table S5*). During a one-year follow-up, non-enrolled patients initiating treatment were less likely adhere to all four drugs after adjustment for factors that should affect adherence (Model 1, *Table 3*). However, these differences were no longer significant after further adjustment for demographics, comorbidities and revascularisation (Model 3). DAPT was initiated or continued in 36.5% of non-enrolled vs. 73.6% of enrolled patients, and non-enrolled patients were also less likely to remain on DAPT beyond 3 months in all models (*Table 3*; details in Supplementary material online, *Table S8*). In our supporting analysis, non-enrolled patients with CKD were less likely to initiate or continue treatment than those without CKD, despite adjustment for factors that should affect treatment initiation (Model 1, Supplementary material online, *Table S6*). However, first-year adherence was significantly lower only for RASi/ARNI (see Supplementary material online, *Table S7*).

**Table 2:** Associations of coronary interventions and short-term mortality in non-enrolled vs. enrolled patients

	All *n* = 47 342	Enrolled *n* = 41 229	Non-enrolled *n* = 6113	Adjusted odds ratio^a^
**Coronary angiography**	31 947 (68%)	30 773 (75%)	1174 (19%)	0.14 (0.13–0.15)
**Revascularisation**				
PCI	24 346 (51%)	23 751 (58%)	595 (9.7%)	0.14 (0.13–0.15)
CABG	2243 (4.7%)	2067 (5.0%)	176 (2.9%)	1.08 (0.91–1.27)
In-hospital death	3700 (7.8%)	2408 (5.8%)	1292 (21%)	2.02 (1.85–2.20)

	All *n* = 43 642	Enrolled *n* = 38 821	Non-enrolled *n* = 4821	Adjusted hazard ratio^b^
**Death within 90 days after discharge (events)**	1907	1359	548	1.14 (1.02–1.26)

PCI, percutaneous coronary intervention; CABG, coronary-artery bypass graft.

^a^Logistic regression showing odds ratio for comparing non-enrolled vs. enrolled (reference) patients, adjusted for demographic factors (age, gender, cohabitating, levels of education and income, inclusion year), comorbidities (acute coronary syndrome or other ischemic heart disease, atrial fibrillation, congestive heart failure, chronic obstructive pulmonary disease, diabetes mellitus, hypertension, malignancy ≤3 years, peripheral artery disease, stroke, estimated glomerular filtration rate category, mild cognitive impairment or dementia, and revascularisation (percutaneous coronary intervention or coronary artery bypass graft).

^b^Cox regression of 90-day mortality comparing non-enrolled vs. enrolled patients (reference), censored for <90-day observation or end of follow-up, adjusted as above, with comorbidities recalculated at discharge.

**Table 3:** Use of guideline-recommended drugs and adherence in non-enrolled vs. enrolled patients

Initiation/continuation	ASA	BB	RASi/ARNI	Statins	DAPT >3 months^b^
** Non-enrolled**	3295	3565	2385	2175	741/3924 (18.9%)
** Enrolled**	32 326	32 631	28 082	31 830	18 962/35 287 (53.6%)
**Crude HR** ^a^ **/OR** ^b^	0.60 (0.58–0.62)	0.69 (0.66–0.71)	0.52 (0.50–0.55)	0.34 (0.33–0.36)	0.20 (0.19–0.22)
**Adjusted for**					
** Model 1**	0.74 (0.71–0.76)	0.69 (0.66–0.71)	—	—	0.19 (0.17–0.20)
** Model 2**	0.85 (0.81–0.88)	0.80 (0.77–0.83)	0.63 (0.61–0.66)	0.48 (0.46–0.50)	0.31 (0.28–0.34)
** Model 3**	0.88 (0.84–0.92)	0.87 (084.-0.90)	0.73 (0.70–0.76)	0.59 (0.56–0.61)	0.54 (0.49–0.59)

Adherence	ASA	BB	RASi/ARNI	Statins
** Non-enrolled**	2867/3295 (87.0%)	3161/3565 (88.7%)	2072/2385 (86,9%)	1901/2175 (87.4%)
** Enrolled**	29 090/32 326 (90.0%)	29 577/32 631 (90.6%)	25 342/28 082 (90.2%)	29 020/31 830 (91.2%)
** All**	31 957/35 621 (89.7%)	32 738/36 196 (90.4%)	27 414/30 467 (90.0%)	30 921/34 005 (90.1%)
**Crude OR** ^c^	0.75 (0.67–0.83)	0.81 (0.72–0.90)	0.72 (0.63–0.81)	0.67 (0.59–0.77)
**Adjusted for**				
** Model 1**	0.82 (0.73–0.93)	0.81 (0.73–0.90)	—	—
** Model 2**	1.01 (0.90–1.15)	0.82 (0.74–0.93)	0.84 (0.74–0.96)	0.84 (0.73–0.96)
** Model 3**	1.13 (0.99–1.28)	0.93 (0.83–1.05)	0.91 (0.80–1.04)	1.02 (0.89–1.18)

ASA, acetylsalicylic acid; BB, beta-blocker; RASi, RASi: renin angiotensin system inhibitor; ARNI, angiotensin receptor-neprilysin inhibitor; DAPT, dual antiplatelet therapy. HR, hazard ratio; OR, odds ratio.

^a^Cause-specific Cox regression of drug dispensation within 90 days, comparing non-enrolled (*n* = 4821) vs. enrolled patients (reference, *n* = 38 821), censored at death or end of follow-up.

^b^Logistic regression of DAPT use >3 months comparing non-enrolled vs. enrolled patients. Patients who died or were lost to follow-up within 120 days post-discharge (*n* = 4344) were excluded from the analysis of use of DAPT.

^c^Logistic regression of drug adherence (proportion of days covered, PDC, ≥80%) during the first year of treatment, comparing non-enrolled vs. enrolled patients (reference). PDC from first dispense to 365 days, death or end of follow-up.

Model 1: Adjusted for absolute/relative contraindications for ASA: other antiplatelet/anticoagulant within 90 days, prior or in-hospital bleeding; for BB: atrioventricular block without a pacemaker.

Model 2: Model 1 + demographic factors and comorbidities (same as *Table 2* except for inclusion year).

Model 3: Model 2 + revascularisation (percutaneous coronary intervention or coronary artery bypass graft).

### Short- and long-term clinical outcomes

Overall, 7.8% of patients with myocardial infarction died in-hospital. The in-hospital mortality was 21% among non-enrolled patients compared to 5.8% among those enrolled (*P* < 0.001). After adjustment, non-enrolled patients had two-fold increased odds of in-hospital death and were significantly more likely to die within 90 days post-discharge (*Table 2*).

**Figure 2:**
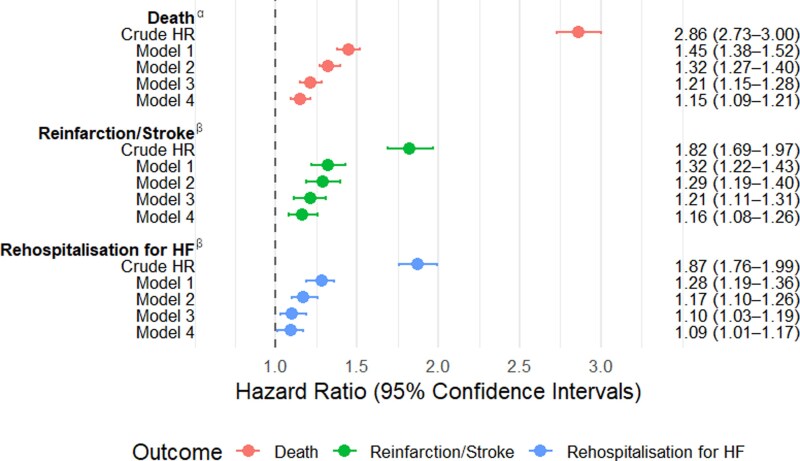
Clinical outcomes after 90 days from discharge in non-enrolled vs. enrolled patients. HF: heart failure. Hazard ratios (HR) comparing non-enrolled vs. enrolled patients (reference) from 90 days post-discharge. ^α^Cox proportional regression censored at the end of follow-up. [events = 12 373; follow-up ≤ 5748 days; median 2379 (1020–3947)]. ^β^Cause-specific Cox regression, censored at migration, death, or end of follow-up. Model 1: Adjusted for demographic variables (age, gender, cohabitating, levels of education and income, and inclusion year). Model 2: Model 1 + comorbidities (acute coronary syndrome or other ischemic heart disease, atrial fibrillation, congestive heart failure, chronic obstructive pulmonary disease, diabetes mellitus, hypertension, malignancy in 3 previous years, peripheral artery disease, stroke, estimated glomerular filtration rate stage, mild cognitive impairment or dementia). Model 3: Model 2 + revascularisation (percutaneous coronary intervention or coronary artery bypass graft). Model 4: Model 3 + use of secondary prevention treatment (antiplatelet/anticoagulant, beta-blockers, statins and renin-angiotensin system-acting drugs) within 90 days after discharge.

After exclusion of patients whose observation period ended due to in-hospital death, myocardial infarction re-hospitalisation within 30–90 days, or loss to follow-up within 90 days after discharge, 40 215 hospitalisations remained for the long-term mortality analysis. Median follow-up was 6.5 years (IQR 2.8–10.8; maximum 15.7 years), during which 12 373 patients died. After adjusting for demographics and comorbidities, non-enrollment was associated with higher mortality (HR 1.32, 95% CI 1.27–1.40; *Figure 2*). Further adjustment for invasive procedures and secondary prevention medication (Model 4) attenuated but did not eliminate the association (HR 1.15, 95% CI 1.09–1.21). After excluding emigrants from Stockholm, 40 127 hospitalisations remained for non-fatal event analysis. In adjusted cause-specific Cox models, censoring for death, non-enrollment was associated with higher risks of heart failure re-hospitalisation and the combined endpoint of reinfarction or stroke. Sensitivity analysis restricted to incident cases showed consistent results (see Supplementary material online, *Table S9*).

In our supporting analysis among older adults (see Supplementary material online, *Table S10*), the interaction between frailty risk and non-enrollment was significantly associated with long-term mortality in the unadjusted results and after adjusting for demographics, but this association weakened and became non-significant after adjusting for comorbidities, revascularisation, and secondary prevention medications, indicating that the association between enrollment and long-term mortality differs between frailty risk groups but is attenuated when taking comorbidities and treatment into account.

## Discussion

This study identifies important differences in characteristics, clinical care, and outcomes between patients enrolled in the SWEDEHEART registry and those not enrolled after a first or recurrent acute myocardial infarction. Approximately one in ten myocardial infarction cases were not enrolled in SWEDEHEART; these patients were older, had poorer kidney function, and carried a higher comorbidity burden. They were less likely to undergo invasive procedures or receive guideline-recommended secondary prevention medications after discharge. Non-enrollment was associated with increased short- and long-term mortality even after extensive adjustments. This study thus identifies a non-negligible population in need of improved care. Enrollment in SWEDEHEART entails the use of a structured protocol for data collection and clinical documentation, the results of which are published in an annual quality index for each affiliated hospital. Our findings support the value of structured care models and benchmarking for improving patient outcomes through closer monitoring and better treatment.

Over time, SWEDEHEART has expanded its national coverage from approximately 50–60% to around 90%, with improved inclusion across regions and hospital wards.^18,19^ However, national coverage among patients aged 80 years or older remains lower (around 75%) and has shown little improvement over time, with substantial variability between hospitals.^20^ Consistent with these trends, 13% of myocardial infarction cases in our study were not enrolled. These individuals were generally older, more often female, and exhibited greater frailty and comorbidity.^18,19,21^ It is plausible that a subset of these non-enrolled patients had type 2 myocardial infarctions,^22^ as suggested by higher proportions with elevated CRP and low haemoglobin levels. Notably, over half of the non-enrolled group had CKD, a particularly complex phenotype associated with increased risks of in-hospital mortality,^23^ recurrent events,^24^ and long-term mortality.^25^

Non-enrolled patients underwent coronary angiography and PCI less frequently. This may partly reflect their higher in-hospital mortality or the logistical need for transfer to a SWEDEHEART-affiliated ward for angiography. Furthermore, non-enrolled patients were less likely to initiate or continue guideline-recommended pharmacotherapy, underscoring a sizable subgroup receiving suboptimal secondary prevention. These findings are consistent with prior work showing that patients enrolled in quality registries generally receive more comprehensive care, both perceived^26^ and measured objectively.^1^ For instance, registry studies in diabetes^27^ and heart failure^28^ have similarly found that non-enrolled patients were less often prescribed evidence-based medications and experienced poorer clinical outcomes. We also observed that non-enrolled patients exhibited lower treatment adherence, although sequential multivariable analyses suggested that this may be partly explained by their higher comorbidity burden.

Non-enrolled participants had approximately two-fold higher odds of in-hospital death and a higher long-term mortality compared with enrolled participants. These differences were partly explained by lower rates of revascularisation and less frequent use of secondary prevention therapies, highlighting care gaps that may be amenable to intervention. Nonetheless, given the advanced age, substantial disease burden, and possibly higher proportion of type 2 myocardial infarctions among non-enrolled patients, it is plausible that not all would derive meaningful benefit from invasive or intensive treatment strategies. Identifying which patients are most likely to benefit from such interventions remains a major clinical challenge. Although the SENIOR-RITA trial found no benefit of PCI among older adults with NSTEMI,^29^ observational studies have reported survival advantages in both older populations^30^ and in patients with CKD.^31^ Similarly, the use of guideline-recommended secondary preventive drugs has been associated with improved outcomes also in patients with type 2 myocardial infarctions,^32^ and a recent review supports the use of aspirin and statins in selected patients among them.^33^

Collectively, our findings provide indirect support for the hypothesis that structured care models, including enrollment in quality registries^1,26-28^ can enhance the quality of care by promoting more consistent delivery and follow-up of treatment. The standardized data collection protocols used in registries may aid clinical decision-making by serving as reminders for clinicians^34^ and encouraging adherence to evidence-based practices.^35^ In addition, they facilitate systematic tracking of quality indicators and enable performance feedback, fostering continuous improvement in care.^36^

Our study has limitations. First, as an observational analysis, it cannot establish causality. While we identified multiple indicators of care quality, unmeasured factors such as comorbid conditions, overall illness severity, or incorrectly classified type 2 myocardial infarctions may have influenced clinical decisions. Second, the generalisability of our findings beyond the Stockholm region should be interpreted with caution. Third, we identified moderate-to-severe CKD based on a single eGFR measurement. Finally, outcome and comorbidity ascertainment relied on ICD codes, which, despite their high diagnostic validity,^37^ remain subject to potential misclassification bias.

In conclusion, we identified a vulnerable subgroup of myocardial infarction patients who were not enrolled in the national quality registry, which received less optimal acute care, less secondary preventive medications, and experienced worse outcomes. Whether these disparities reflect stricter clinical practices at registry-affiliated centres or the greater burden of illness in non-enrolled patients remains uncertain, but non-enrollment remained associated with increased short- and long-term mortality even after extensive adjustments. Clinically, our findings highlight important care gaps that warrant targeted interventions and reinforce the value of structured care models. Active hospital-level efforts should be made to improve inclusion of non-cardiology wards, including geriatric wards, through simplified registry workflows and structured cardiology support. Finally, this study underscores the need for caution when generalising findings from registries with incomplete coverage and illustrates the value of analyses based on comprehensive health system data.

## Supplementary Material

qcag023_Supplementary_Data

## Data Availability

Data available on reasonable request to Prof. Carrero (juan.jesus.carrero@ki.se) for research compliant with the General Data Protection Regulation and ethical regulations.
