# Supplementary material for: Characteristics, clinical management and outcomes of patients with acute myocardial infarction enrolled or not enrolled in a quality registry
Source: Eur Heart J Qual Care Clin Outcomes. 2026 Feb 10;12(4):647–56. doi: 10.1093/ehjqcco/qcag023 (PMC13288745; doi:10.1093/ehjqcco/qcag023)
Supplement: qcag023_Supplementary_Data [file qcag023_supplementary_data.docx]

## **Table S1**. Definitions and categorizations of covariates, procedures and clinical outcomes

| **Variable** | **Categorization** | **ICD 10 codes** | **Data source** |
| --- | --- | --- | --- |
| ***Demographics and socioeconomic status*** | | | |
| Sex | Man; woman |  | VAL |
| Age, years | Continuous |  | VAL |
| Age category, years | 18-49; 50-64; 65-79; ≥80 |  | VAL |
| Civil status (married/cohabiting) | Yes/No |  | Statistics Sweden |
| Education level | Compulsory school/ Secondary school/ University |  | Statistics Sweden |
| Disposable income | Quartiles |  | Statistics Sweden |
| ***Laboratory variables*** | | | |
| eGFR category (mL/min/1.73 m2) | ≥60 / 30 - 59 / <30 |  | SCREAM |
| Any Hemoglobin <90 g/L | Yes/No |  | SCREAM |
| Any CRP >80 mg/L | Yes/No |  | SCREAM |
| ***Condition relevant to the index event*** | | | |
| Infarction type |  |  |  |
| Transmural infarction | Yes/No | I21.0-I21.3 | VAL |
| Subendocardial infarction | Yes/No | I21.4 | VAL |
| Unspecified | Yes/No | I21.9 | VAL |
| Calender year | 2006 / 2007 / 2008 / 2009 / 2010 / 2011 / 2012 / 2013 / 2014 /  2015 / 2016 / 2017 /  2018 / 2019 / 2020 /  2021 |  | VAL |
| Care at cardiology unit | Yes/No | 231* | VAL |
| Discharged from geriatric unit | Yes/No | 240, 241, 246* | VAL |
| Previous or in-hospital bleeding | Yes/No | I60, I61, I62, D629, I850, K226, K250, K252, K254, K256, K260, K262, K264, K266, K270, K272, K274, K276, K280, K282, K284, K286, K290, K625, K920, K921, K922 | VAL |
| ***Comorbidities at admission*** | | | |
| Acute coronary syndrome | Yes/No | I200,I21-I22 | VAL |
| Other ischemic heart disease | Yes/No | I201, I208, I209, I24, I25 | VAL |
| Intracranial bleeding | Yes/No | I60-I62 | VAL |
| Gastrointestinal bleeding | Yes/No | K92.9, K22.6, K25.0, K25.2, K25.4, K25.6, K26.0, K26.2, K26.4, K26.6, K27.0, , K27.2, K27.4, K27.6, I98.3, I85.0 | VAL |
| Atrial fibrillation | Yes/No | I48 | VAL |
| AV-block of any degree | Yes/No | I44.0-I44.3 | VAL |
| Congestive heart failure | Yes/No | I50, I42-143.8, I11.0, I13.0, I13.2 I50, I25.5 | VAL |
| Chronic respiratory disease | Yes/No | I27.8, I27.9, J40 -J47, J60-J67, J68.4, J70.1, J70.3 | VAL |
| Chronic obstructive pulmonary disorder | Yes/No | J44 | VAL |
| Diabetes mellitus | Yes/No | E10-E14 | VAL |
| Hypertension | Yes/No | I10.0-I15.9 | VAL |
| Malignancy in previous 3 years | Yes/No | C00-C97 | VAL |
| Peripheral arterial disease | Yes/No | I70, I73.9, I74, I77 | VAL |
| Stroke | Yes/No | I61, I63, I64 | VAL |
| End stage kidney disease | Yes/No | N18.5 | VAL |
| Dialysis (at any time) | Yes/No | Z49.1, Z49.2 | VAL |
| Mild cognitive impairment | Yes/No | F06.7, R41.8A | VAL |
| Dementia | Yes/No | F00-F03, F05.1, G30, G31.1 | VAL |
| ***Additional variables for calculating Charlson comorbidity index*** | | | |
| Rheumatic disease | Yes/No | M05, M06, M31.5, M32-M34, M35.1, M35.3, M36.0 | VAL |
| Peptic ulcer disease | Yes/No | K25- K28 | VAL |
| Diabetes without chronic complication | Yes/No | E10.0, E10.1, E10.6, E10.8, E10.9, E11.0, E11.1, E11.6, E11.8, E11.9, E12.0, E12.1, E12.6, E12.8, E12.9, E13.0, E13.1, E13.6, E13.8, E13.9, E14.0, E14.1, E14.6, E14.8, E14.9 | VAL |
| Diabetes with chronic complication | Yes/No | E10.2 - E10.5, E10.7, E11.2 - E11.5, E11.7, E12.2 - E12.5, E12.7, E13.2 - E13.5, E13.7, E14.2 - E14.5, E14.7 | VAL |
| Hemiplegia or paraplegia | Yes/No | G04.1, G11.4, G80.1, G80.2, G81, G82, G83.0 - G83.4, G83.9 | VAL |
| Renal disease | Yes/No | I12.0, I13.1, N03.2-N03.7, N05.2-N05.7, N18, N19, N25.0, Z49.0-Z49.2, Z94.0, Z99.2 | VAL |
| Mild liver disease | Yes/No | B18, K70.0-K70.3, K70.9, K71.3-K71.5, K71.7, K73, K74, K76.0, K76.2-K76.4, K76.8, K76.9, Z94.4 | VAL |
| Moderate or severe liver disease | Yes/No | I85.0, I85.9, I86.4, I98.2, K70.4, K71.1, K72.1, K72.9, K76.5, K76.6, K76.7 | VAL |
| Metastatic solid tumour | Yes/No | C77-C80 | VAL |
| Any malignancy, including lymphoma and leukemia, except malignant neoplasms of skin | Yes/No | C00-C26, C30- C34, C37- C41, C43, C45-C58, C60-C76, C81- C85, C88, C90- C97 | VAL |
| ***Medications at admission*** |  | **ATC codes** |  |
| Acetylsalicylic acid | Yes/No | B01AC06 | NPDR |
| Other antiplatelet drug | Yes/No | B01AC except B01AC06 | NPDR |
| Oral anticoagulants (Warfarin, NOAC) | Yes/No | B01AA03, B01AF | NPDR |
| Beta-blockers | Yes/No | C07 | NPDR |
| Calcium-channel blockers | Yes/No | C08 | NPDR |
| RASi/ARNI | Yes/No | C09 | NPDR |
| Aldosterone antagonists | Yes/No | C03D | NPDR |
| Statins | Yes/No | C10AA, C10BA | NPDR |
| Ezetimibe (monotherapy or combined) | Yes/No | C10AX09, C10BA02, C10BA05 | NPDR |
| ***Invasive procedures*** |  | **Procedure codes** |  |
| Coronary angiography |  | AF037 | VAL |
| Revascularization |  |  |  |
| Percutaneous coronary intervention (PCI) |  | FNG02, FNG05 | VAL |
| Coronary artery bypass graft (CABG) |  | FNA00, FNA10, FNC10, FNC10, FNC20, FNC30, FNC40 | VAL |
| ***Clinical outcomes*** |  | **ICD codes** |  |
| In-hospital death |  | Any code | Population register |
| All-cause mortality after discharge |  | Any code | Population register |
| Myocardial infarction |  | I21, I22, I23 | VAL |
| Ischemic stroke |  | H341, G45-46, I60- I61, I63, I64 | VAL |
| Hospital admission due to heart failure |  | I50 (primary or second position) | VAL |

*Administrative codes (Kombika) identifying the associated medical specialty

## **Table S2**. Sensitivity analysis in subgroup of incident cases: Characteristics at admission according to quality register enrolment

| **Variable** | **All**  N=40,935 | **Enrolled**  N=35,903 | **Non-enrolled**  N=5,032 | **P-value** |
| --- | --- | --- | --- | --- |
| **Demographics and socioeconomic status** | | | | |
| Male | 26,016 (64%) | 23,673 (66%) | 2,343 (47%) | <0.001 |
| Age, years, median (IQR) | 72 (62, 82) | 71 (61, 80) | 84 (75, 89) | <0.001 |
| Age category, years |  |  |  | <0.001 |
| 18-49 | 2,585 (6.3%) | 2,509 (7.0%) | 76 (1.5%) |  |
| 50-64 | 10,531 (26%) | 10,056 (28%) | 475 (9.4%) |  |
| 65-79 | 15,366 (38%) | 14,127 (39%) | 1,239 (25%) |  |
| 80+ | 12,453 (30%) | 9,211 (26%) | 3,242 (64%) |  |
| Cohabitating | 0,987 (51%) | 19,147 (53%) | 1,840 (37%) | <0.001 |
| Education level |  |  |  | <0.001 |
| Compulsory school | 12,798 (31%) | 10,715 (30%) | 2,083 (41%) |  |
| Secondary school | 16,476 (40%) | 14,674 (41%) | 1,802 (36%) |  |
| University | 10,317 (25%) | 9,476 (26%) | 841 (17%) |  |
| Missing | 1,344 (3.3%) | 1,038 (2.9%) | 306 (6.1%) |  |
| Disposable Income (quartiles) |  |  |  | <0.001 |
| 1 (lowest) | 9,871 (24%) | 8,386 (23%) | 1,485 (30%) |  |
| 2 | 9,820 (24%) | 8,243 (23%) | 1,577 (31%) |  |
| 3 | 10,268 (25%) | 9,106 (25%) | 1,162 (23%) |  |
| 4 (highest) | 10,846 (26%) | 10,049 (28%) | 797 (16%) |  |
| **Laboratory variables** | | | | |
| eGFR category (mL/min/1.73 m²) |  |  |  | <0.001 |
| ≥60 | 27,066 (66%) | 25,049 (70%) | 2,017 (40%) |  |
| 30-59 | 9,521 (23%) | 7,671 (21%) | 1,850 (37%) |  |
| <30 | 2,351 (5.7%) | 1,640 (4.6%) | 711 (14%) |  |
| Missing | 1,997 (4.9%) | 1,543 (4.3%) | 454 (9.0%) |  |
| Any Hemoglobin <90 g/L (N=43,933) | 2,889 (7.6%) | 2,410 (7.2%) | 479 (11%) | <0.001 |
| Any CRP >80 mg/L (N=42,314) | 8,585 (24%) | 7,181 (22%) | 1,404 (33%) | <0.001 |
| **Condition relevant to the index event** | | | | |
| Infarction type |  |  |  | <0.001 |
| Transmural infarction | 11,242 (27%) | 10,769 (30%) | 473 (9.4%) |  |
| Subendocardial infarction | 20,106 (49%) | 18,027 (50%) | 2,079 (41%) |  |
| Unspecified | 9,587 (23%) | 7,107 (20%) | 2,480 (49%) |  |
| Calendar year |  |  |  | <0.001 |
| 2006-2009 | 12,207 (30%) | 8,818 (25%) | 3,389 (67%) |  |
| 2010-2013 | 10,229 (25%) | 9,377 (26%) | 852 (17%) |  |
| 2014-2017 | 9,637 (24%) | 9,205 (26%) | 432 (8.6%) |  |
| 2018-2021 | 8,862 (22%) | 8,503 (24%) | 359 (7.1%) |  |
| Care at cardiology unit | 32,452 (79%) | 30,323 (84%) | 2,129 (42%) | <0.001 |
| Discharged to home from geriatric unit | 5,143 (13%) | 3,688 (10%) | 1,455 (29%) | <0.001 |
| **Comorbidities at admission** | | | | |
| Previous acute coronary syndrome | 10,315 (25%) | 9,199 (26%) | 1,116 (22%) | <0.001 |
| Other ischemic heart disease | 9,628 (24%) | 7,962 (22%) | 1,666 (33%) | <0.001 |
| Intracranial bleeding | 671 (1.6%) | 559 (1.6%) | 112 (2.2%) | <0.001 |
| Gastrointestinal bleeding | 779 (1.9%) | 651 (1.8%) | 128 (2.5%) | <0.001 |
| Atrial fibrillation | 5,642 (14%) | 4,383 (12%) | 1,259 (25%) | <0.001 |
| AV-block of any degree | 771 (1.9%) | 595 (1.7%) | 176 (3.5%) | <0.001 |
| Congestive heart failure | 6,477 (16%) | 4,765 (13%) | 1,712 (34%) | <0.001 |
| Chronic obstructive pulmonary disorder | 4,504 (11%) | 3,745 (10%) | 759 (15%) | <0.001 |
| Other chronic respiratory disease | 5,033 (12%) | 4,380 (12%) | 653 (13%) | 0.12 |
| Diabetes mellitus | 10,607 (26%) | 9,303 (26%) | 1,304 (26%) | >0.9 |
| Hypertension | 26,732 (65%) | 23,387 (65%) | 3,345 (66%) | 0.062 |
| Malignancy in previous 3 years | 4,557 (11%) | 3,838 (11%) | 719 (14%) | <0.001 |
| Peripheral arterial disease | 3,785 (9.2%) | 3,064 (8.5%) | 721 (14%) | <0.001 |
| Stroke | 3,691 (9.0%) | 2,915 (8.1%) | 776 (15%) | <0.001 |
| End stage kidney disease | 325 (0.8%) | 284 (0.8%) | 41 (0.8%) | 0.9 |
| Dialysis (at any time) | 337 (0.8%) | 276 (0.8%) | 61 (1.2%) | 0.001 |
| Mild cognitive impairment | 1,781 (4.4%) | 1,344 (3.7%) | 437 (8.7%) | <0.001 |
| Dementia | 1,937 (4.7%) | 1,328 (3.7%) | 609 (12%) | <0.001 |
| **Comorbidity/Frailty Risk scores** | | | | |
| Charlson Comorbidity Index, median (IQR) | 1 (0, 4) | 1 (0, 4) | 2 (1, 4) | <0.001 |
| Hospital Frailty Risk Score |  |  |  | <0.001 |
| Low Risk (<5) | 22,150 (54%) | 20,044 (56%) | 2,106 (42%) |  |
| Intermediate Risk (5-15) | 13,942 (34%) | 11,956 (33%) | 1,986 (39%) |  |
| High Risk (>15) | 4,843 (12%) | 3,903 (11%) | 940 (19%) |  |
| **Medications at admission** | | | | |
| Acetylsalicylic acid | 11,599 (28%) | 9,418 (26%) | 2,181 (43%) | <0.001 |
| Other antiplatelet drug | 2,054 (5.0%) | 1,691 (4.7%) | 363 (7.2%) | <0.001 |
| Oral anticoagulants (Warfarin, DOAC) | 2,827 (6.9%) | 2,393 (6.7%) | 434 (8.6%) | <0.001 |
| Beta-blockers | 13,723 (34%) | 11,595 (32%) | 2,128 (42%) | <0.001 |
| RASi/ARNI | 15,492 (38%) | 13,657 (38%) | 1,835 (36%) | 0.031 |
| Aldosterone antagonists | 1,780 (4.3%) | 1,324 (3.7%) | 456 (9.1%) | <0.001 |
| Statins | 9,962 (24%) | 8,848 (25%) | 1,114 (22%) | <0.001 |
| Ezetimibe | 447 (1.1%) | 416 (1.2%) | 31 (0.6%) | <0.001 |

IQR: interquartile range; NT-proBNP: N-terminal prohormone of brain natriuretic peptide; CRP: C-reactive protein; AV-block: atrioventricular-block; DOAC: direct oral anticoagulant; RASi: renin angiotensin system inhibitor; ARNI: angiotensin receptor-neprilysin inhibitor;

## **Table S3**. Sensitivity analysis by period: Hazard Ratios of using guideline recommended therapies in participants enrolled or non-enrolled in SWEDEHEART during 2006-2009

|  | **ASA** | **BB** | **RASi/ARNI** | **Statins** |
| --- | --- | --- | --- | --- |
| **Initiation and continuation** |  |  |  |  |
| **Non-enrolled** | 2,240 | 2,400 | 1,531 | 1,450 |
| **Enrolled** | 7,740 | 7,946 | 6,266 | 7,175 |
| **Crude HR** | 0.60 (0.57-0.63) | 0.65 (0.62-0.68) | 0.55 (0.52-0.58) | 0.39 (0.36-0.41) |
| **Adjusted HR** |  |  |  |  |
| **Model 1** | 0.73 (0.70-0.77) | 0.65 (0.62-0.68) | - | - |
| **Model 2** | 0.84 (0.79-0.88) | 0.81 (0.77-0.85) | 0.66 (0.62-0.70) | 0.58 (0.54-0.61) |
| **Model 3** | 0.87 (0.82-0.91) | 0.86 (0.81-0.90) | 0.72 (0.68-0.76) | 0.65 (0.61-0.69) |

ASA: acetylsalicylic acid; BB: beta-blocker; RASi: RASi: renin angiotensin system inhibitor; ARNI: angiotensin receptor-neprilysin inhibitor.

Cause-specific Cox regression showing hazard ratio for drug dispensation within 90 days comparing non-enrolled (N=3,253) versus enrolled (reference, N=9,156) patients. Censored for death or end of follow up.

Model 1: Adjusted for absolute/relative contraindications for ASA: other antiplatelet/anticoagulant initiated within 90 days, previous or in-hospital bleeding; for BB: history of AV-block of any degree without pacemaker.

Model 2: In addition to factors of model 1, also adjusted for demographic factors (age, gender, cohabitating, educational level, income level), comorbidities (acute coronary syndrome or other ischemic heart disease, atrial fibrillation, congestive heart failure, chronic obstructive pulmonary disease, diabetes mellitus, hypertension, malignancy in 3 previous years, peripheral artery disease, stroke, estimated glomerular filtration rate stage, mild cognitive impairment or dementia).

Model 3: In addition to factors in model 2, also adjusted for revascularization (percutaneous coronary intervention or coronary artery bypass graft).

## **Table S4**. Sensitivity analysis by period: Hazard Ratios of using guideline recommended therapies in participants enrolled or non-enrolled in SWEDEHEART during 2010-2021

|  | **ASA** | **BB** | **RASi/ARNI** | **Statins** |
| --- | --- | --- | --- | --- |
| **Initiation or continuation** |  |  |  |  |
| **Non-enrolled** | 1,038 | 1,141 | 839 | 715 |
| **Enrolled** | 24,504 | 24,592 | 21,732 | 24,591 |
| **Crude hazard ratio** | 0.57 (0.54-0.60) | 0.67 (0.64-0.71) | 0.58 (0.54-0.61) | 0.33 (0.31-0.36) |
| **Adjusted for** |  |  |  |  |
| **Model 1** | 0.72 (0.68-0.76) | 0.67 (0.64-0.71) | - | - |
| **Model 2** | 0.86 (0.80-0.91) | 0.78 (0.73-0.82) | 0.68 (0.64-0.73) | 0.47 (0.43-0.50) |
| **Model 3** | 0.89 (0.83-0.95) | 0.85 (0.80-0.90) | 0.80 (0.75-0.86) | 0.59 (0.55-0.64) |

ASA: acetylsalicylic acid; BB: beta-blocker; RASi: RASi: renin angiotensin system inhibitor; ARNI: angiotensin receptor-neprilysin inhibitor.

Cause-specific Cox regression showing hazard ratio for drug dispensation within 90 days comparing non-enrolled (N=1,568) versus enrolled (reference, N=29,665) patients. Censored for death or end of follow up.

Model 1: Adjusted for absolute/relative contraindications for ASA: other antiplatelet/anticoagulant initiated within 90 days, previous or in-hospital bleeding; for BB: history of AV-block of any degree without pacemaker.

Model 2: In addition to factors of model 1, also adjusted for demographic factors (age, gender, cohabitating, educational level, income level), comorbidities (acute coronary syndrome or other ischemic heart disease, atrial fibrillation, congestive heart failure, chronic obstructive pulmonary disease, diabetes mellitus, hypertension, malignancy in 3 previous years, peripheral artery disease, stroke, estimated glomerular filtration rate stage, mild cognitive impairment or dementia)

Model 3: In addition to factors in model 2, also adjusted for revascularization (percutaneous coronary intervention or coronary artery by-pass graft).

## **Table S5**. Supporting analysis in subgroup of incident cases: Hazard Ratios of using guideline recommended therapies in participants enrolled or not in SWEDEHEART

|  | **ASA** | **BB** | **RASi/ARNI** | **Statins** |
| --- | --- | --- | --- | --- |
| **Initiation and continuation** |  |  |  |  |
| **Non-enrolled** | 2,736 | 2,933 | 1,894 | 1,725 |
| **Enrolled** | 28,886 | 28,677 | 24,626 | 28,315 |
| **Crude hazard ratio** | 0.57 (0.55-0.59) | 0.67 (0.65-0.69) | 0.49 (0.47-0.52) | 0.31 (0.30-0.33) |
| **Adjusted for** |  |  |  |  |
| **Model 1** | 0.72 (0.59-0.75) | 0.67 (0.65-0.69) | - | - |
| **Model 2** | 0.84 (0.81-0.88) | 0.80 (0.77-0.83) | 0.60 (0.57-0.63) | 0.45 (0.43-0.58) |
| **Model 3** | 0.87 (0.83-0.91) | 0.88 (0.84-0.91) | 0.70 (0.67-0.74) | 0.56 (0.53-0.59) |

ASA: acetylsalicylic acid; BB: beta-blocker; RASi: RASi: renin angiotensin system inhibitor; ARNI: angiotensin receptor-neprilysin inhibitor.

Cause-specific Cox regression showing hazard ratio for drug dispensation within 90 days comparing non-enrolled (N=3,947) versus enrolled (reference, N=33,857) patients. Censored for death or end of follow up.

Model 1: Adjusted for absolute/relative contraindications for ASA: other antiplatelet/anticoagulant initiated within 90 days, previous or in-hospital bleeding; for BB: history of AV-block of any degree without pacemaker.

Model 2: In addition to factors of model 1, also adjusted for demographic factors (age, gender, cohabitating, educational level, income level), comorbidities (acute coronary syndrome or other ischemic heart disease, atrial fibrillation, congestive heart failure, chronic obstructive pulmonary disease, diabetes mellitus, hypertension, malignancy in 3 previous years, peripheral artery disease, stroke, estimated glomerular filtration rate stage, mild cognitive impairment or dementia).

Model 3: In addition to factors in model 2, also adjusted for revascularization (percutaneous coronary intervention or coronary artery bypass graft).

## **Table S6**. Supporting analysis in the subgroup of non-enrolled participants: Guideline-recommended drug use in participants with and without CKD

|  | **ASA** | **BB** | **RASi/ARNI** | **Statins** |
| --- | --- | --- | --- | --- |
| **Initiation or continuation** |  |  |  |  |
| **No CKD** | 1,486 | 1,560 | 1,089 | 1,100 |
| **CKD** | 1,477 | 1,666 | 1,085 | 857 |
| **Crude HR** | 0.74 (0.68-0.79) | 0.81 (0.76-0.87) | 0.80 (0.74-0.87) | 0.57 (0.52-0.63) |
| **Adjusted HR** |  |  |  |  |
| **Model 1** | 0.78 (0.72-0.83) | 0.81 (0.76-0.87) | - | - |
| **Model 2** | 0.85 (0.78-0.92) | 0.90 (0.84-0.97) | 0.79 (0.72-0.87) | 0.79 (0.71-0.87) |
| **Model 3** | 0.86 (0.80-0.94) | 0.92 (0.85-0.99) | 0.80 (0.73-0.88) | 0.83 (0.75-0.91) |

ASA: acetylsalicylic acid; BB: beta-blocker; RASi: RASi: renin angiotensin system inhibitor; ARNI: angiotensin receptor-neprilysin inhibitor.

Cause-specific Cox regression showing hazard ratio for drug dispensation within 90 days comparing patients with comparing patients with CKD (eGFR <60 ml/min/1.73 m2, N=2,023) versus no CKD (eGFR ≥60 ml/min/1.73 m2, N=2,355 reference) among non-enrolled patients. Censored for death or end of follow up.

Model 1: Adjusted for absolute/relative contraindications for ASA: other antiplatelet/anticoagulant initiated within 90 days, previous or in-hospital bleeding; for BB: history of AV-block of any degree without pacemaker.

Model 2: In addition to factors of model 1, also adjusted for demographic factors (age, gender, cohabitating, educational level, income level), comorbidities (acute coronary syndrome or other ischemic heart disease, atrial fibrillation, congestive heart failure, chronic obstructive pulmonary disease, diabetes mellitus, hypertension, malignancy in 3 previous years, peripheral artery disease, stroke, mild cognitive impairment or dementia).

Model 3: In addition to factors in model 2, also adjusted for revascularization (percutaneous coronary intervention or coronary artery bypass graft).

## **Table S7**. Supporting analysis in the subgroup of non-enrolled participants: Odds of good adherence to guideline-recommended therapies during first year of use in participants with and without CKD

| **Proportion of Days Covered ≥ 80%** | **ASA** | **BB** | **RASi/ARNI** | **Statins** |
| --- | --- | --- | --- | --- |
| **No CKD** | 1,306/1,489  (87.7%) | 1,394/1,565  (89.1%) | 968/1,095  (88.4%) | 972/1,103  (88.1%) |
| **CKD** | 1,288/1,490  (86.4%) | 1,489/1,683  (88.5%) | 933/1,094  (85.3%) | 742/864  (85.9%) |
| **All** | 2,594/2,979  (87.1%) | 2,883/3,248  (88.8%) | 1,901/2,189  (86.8%) | 1,714/1,967  (87.1%) |
| **Crude odds ratio** | 0.89 (0.72-1.11) | 0.94 (0.76-1.17) | 0.76 (0.59-0.98) | 0.82 (0.63-1.07) |
| **Adjusted for** |  |  |  |  |
| **Model 1** | 0.89 (0.71-1.13) | 0.94 (0.76-1.17) | - | - |
| **Model 2** | 0.87 (0.60-1.27) | 0.94 (0.73-1.20) | 0.86 (0.65-1.14) | 0.97 (0.72-1.32) |
| **Model 3** | 0.91 (0.62-1.33) | 0.96 (0.75-1.23) | 0.88 (0.68-1.16) | 1.01 (0.74-1.37) |

ASA: acetylsalicylic acid; BB: beta-blocker; RASi: RASi: renin angiotensin system inhibitor; ARNI: angiotensin receptor-neprilysin inhibitor; CKD: chronic kidney disease.

Logistic regression showing odds ratio for drug adherence defined as PDC ≥ 80% comparing patients with CKD (eGFR <60 ml/min/1.73 m2) versus no CKD (eGFR ≥60 ml/min/1.73 m2, reference) in non-enrolled patients with at least one dispensed prescription within 90 days after discharge. PDC was calculated by the ratio of covered days from the day of first dispensed prescription and 365 days ahead or until death/emigration/end of follow up.

Model 1: Adjusted for absolute/relative contraindications for ASA: other antiplatelet/anticoagulant initiated within 90 days, previous or in-hospital bleeding; for BB: history of AV-block of any degree without pacemaker.

Model 2: Additionally adjusted for demographic factors (age, gender, cohabitating, educational level, income level), comorbidities (acute coronary syndrome or other ischemic heart disease, atrial fibrillation, congestive heart failure, chronic obstructive pulmonary disease, diabetes mellitus, hypertension, malignancy in 3 previous years, peripheral artery disease, stroke, mild cognitive impairment or dementia).

Model 3: In addition to factors in model 2, also adjusted for revascularization (percutaneous coronary intervention or coronary artery by-pass graft).

## **Table S8**. Use of DAPT in participants enrolled or non-enrolled in SWEDEHEART

|  | **All  (N=39,298)** | **Enrolled (N=35,369)** | **Non-enrolled (N=3,929)** | **P-value** |
| --- | --- | --- | --- | --- |
| **DAPT status** |  |  |  | <0.001 |
| **No DAPT** | 12,425 (31.6%) | 9,823 (27.8%) | 2,602 (66.2%) |  |
| **DAPT ≤3 months** | 7,170 (18.2%) | 6,584 (18.6%) | 586 (14.9%) |  |
| **DAPT >3 months** | 1,9703 (50.1%) | 18,962 (53.6%) | 741 (18.9%) |  |
|  |  |  |  |  |
| **DAPT >3 months odds ratio** |  | **Enrolled** | **Non-enrolled** |  |
| **Crude** |  | 1.00 (ref) | 0.20 (0.19-0.22) |  |
| **Model 1** |  | 1.00 (ref) | 0.19 (0.17-0.20) |  |
| **Model 2** |  | 1.00 (ref) | 0.31 (0.28-0.34) |  |
| **Model 3** |  | 1.00 (ref) | 0.54 (0.49-0.59) |  |

DAPT: dual antiplatelet therapy.

DAPT was defined as at least one dispensed prescription of acetylsalicylic acid and P2Y12-inhibitor within 90 days after hospital discharge. To allow for variation in packaging sizes, a cut-off of 120 covered days was applied to distinguish between patients treated for 3 months or less and those treated for more than 3 months. Patients that died or were lost to follow up within 120 days after discharge were excluded (N=4,344).

Logistic regression showing odds ratio for comparing non-enrolled versus enrolled patients. Adjusted for comorbidities at day 90 after discharge.

Model 1: Adjusted for absolute/relative contraindications for DAPT: any anticoagulant initiated within 90 days, previous or in-hospital bleeding.

Model 2: In addition to factors of model 1, also adjusted for demographic factors (age, gender, cohabitating, educational level, income level), comorbidities (previous acute coronary syndrome, or other ischemic heart disease, atrial fibrillation, congestive heart failure, chronic obstructive pulmonary disease, diabetes mellitus, hypertension, malignancy in 3 previous years, peripheral artery disease, stroke, estimated glomerular filtration rate, mild cognitive impairment or dementia) and frailty (Hospital Frailty Risk Score).

Model 3: In addition to factors in model 2, also adjusted for revascularization (percutaneous coronary intervention or coronary artery by-pass graft).

## **Table S9**. Sensitivity analysis restricted to incident cases: Clinical outcomes after 90 days from discharge in non-enrolled versus enrolled patients

|  | **Crude HR** | **Model 1** | **Model 2** | **Model 3** | **Model 4** |
| --- | --- | --- | --- | --- | --- |
| **Death^¥^** | 2.88 (2.76-3.00) | 1.40 (1.34–1.47) | 1.27 (1.22–1.33) | 1.16 (1.11–1.22) | 1.10 (1.05–1.15) |
| **Reinfarction/stroke^β^** | 1.82 (1.71-1.95) | 1.32 (1.23-1.42) | 1.29 (1.20-1.39) | 1.21 (1.12-1.30) | 1.16 (1.08-1.25) |
| **Rehospitalisation due to HF^β^** | 1.87 (1.77-1.98) | 1.27 (1.20-1.35) | 1.17 (1.11-1.25) | 1.10 (1.04-1.17) | 1.08 (1.02-1.15) |

Table showing hazard ratios (HR) comparing non-enrolled versus enrolled patients (reference) from 90 days after discharge. HF: Heart failure.

¥ Cox proportional regression censored for end of follow up. Number of events: 13,267. Follow up to 5,748 days, median 2,762 days, interquartile range 1,384; 4,231).

β Cause-specific Cox regression censored for migration, end of follow up or death.

Model 1 – adjusted for demographic variables (age, gender, cohabitating, educational level, income level, year of inclusion).
Model 2 – in addition to Model 1, also adjusted for comorbidities (acute coronary syndrome or other ischemic heart disease, atrial fibrillation, congestive heart failure, chronic obstructive pulmonary disease, diabetes mellitus, hypertension, malignancy in 3 previous years, peripheral artery disease, stroke, estimated glomerular filtration rate stage, mild cognitive impairment or dementia).
Model 3 – in addition to Model 2, also adjusted for revascularization (percutaneous coronary intervention or coronary artery by-pass graft).
Model 4 – in addition to Model 3, also adjusted for initiation/continuation of secondary prevention treatment (antiplatelet/anticoagulant, beta-blockers, statins and RAS-blockers) within 90 days after discharge.

## **Table S10**. Supporting analysis in subgroup of participants aged 75+: All-cause mortality after 90 days from discharge in non-enrolled versus enrolled patients stratified according to Hospital Frailty Risk Score at admission.

|  | **Crude HR** | **Model 1** | **Model 2** | **Model 3** | **Model 4** |
| --- | --- | --- | --- | --- | --- |
| **All patients ≥ 75** N=16,228 | 1.77 (1.68-1.87) | 1.41 (1.33-1.49) | 1.30 (1.23-1.38) | 1.20 (1.14-1.27) | 1.14 (1.08-1.21) |
| **Low Risk (<5**) N=5,948 | 1.74 (1.59-1.89) | 1.29 (1.17-1.41) | 1.23 (1.11-1.45) | 1.14 (1.03-1.25) | 1.07 (0.97-1.18) |
| **Intermediate Risk (5-15)** N=7,070 | 1.97 (1.82-2.13) | 1.43 (1.31-1.56) | 1.34 (1.22-1.46) | 1.24 (1.13-1.36) | 1.19 (1.09-1.31) |
| **High Risk (>15)**  N=3,210 | 1.51 (1.35-1.69) | 1.24 (1.10-1.40) | 1.22 (1.07-1.39) | 1.13 (1.00-1.29) | 1.10 (0.96-1.25) |
| **P for interaction** | <0.001 | 0.01 | 0.07 | 0.13 | 0.15 |

Table showing hazard ratios (HR) comparing non-enrolled versus enrolled patients (reference) from 90 days after discharge until end of follow up.

Cox proportional regression censored for end of follow up. Number of events: 8,534. Follow up to 5,717 days, median 2,166 days, interquartile range 823; 3,828).

Model 1 – adjusted for demographic variables (age, gender, cohabitating, educational level, income level, year of inclusion).
Model 2 – in addition to Model 1, also adjusted for comorbidities (acute coronary syndrome or other ischemic heart disease, atrial fibrillation, congestive heart failure, chronic obstructive pulmonary disease, diabetes mellitus, hypertension, malignancy in 3 previous years, peripheral artery disease, stroke, estimated glomerular filtration rate stage, mild cognitive impairment or dementia).
Model 3 – in addition to Model 2, also adjusted for revascularization (percutaneous coronary intervention or coronary artery by-pass graft).
Model 4 – in addition to Model 3, also adjusted for initiation/continuation of secondary prevention treatment (antiplatelet/anticoagulant, beta-blockers, statins and RAS-blockers) within 90 days after discharge.
